# Consecutive epigenetically-active agent combinations act in *ID1-RUNX3-TET2* and *HOXA* pathways for *Flt3ITD+ve* AML

**DOI:** 10.18632/oncotarget.23655

**Published:** 2017-12-25

**Authors:** Hamid Sayar, Yan Liu, Rui Gao, Mohammad Abu Zaid, Larry D. Cripe, Jill Weisenbach, Katie J. Sargent, Mehdi Nassiri, Lang Li, Heiko Konig, Attaya Suvannasankha, Feng Pan, Rajasubramaniam Shanmugam, Chirayu Goswami, Reuben Kapur, Mingjiang Xu, H. Scott Boswell

**Affiliations:** ^1^ Indiana University Melvin and Bren Simon Cancer Center, Department of Medicine, Hematology/Oncology Division, Indiana University School of Medicine, Indianapolis, IN, USA; ^2^ Department of Hematopathology, Indiana University School of Medicine, Indianapolis, IN, USA; ^3^ Biostatistics and Computational Biology, Indiana University School of Medicine, Indianapolis, IN, USA; ^4^ Department of Pediatrics, Indiana University School of Medicine, Indianapolis, IN, USA; ^5^ Indiana University Health Systems, Indianapolis, IN, USA; ^6^ Veterans Affairs Medical Center, Indianapolis, IN, USA; ^7^ ICMR, National Institute for Research in Tribal Health, Jabalpur, India

**Keywords:** AML, sorafenib, vorinostat, bortezomib, epigenetics

## Abstract

Co-occurrence of *Flt3ITD* and *TET2* mutations provoke an animal model of AML by epigenetic repression of Wnt pathway antagonists, including *RUNX3,* and by hyperexpression of *ID1,* encoding Wnt agonist. These affect *HOXA* over-expression and treatment resistance. A comparable epigenetic phenotype was identified among adult AML patients needing novel intervention. We chose combinations of targeted agents acting on distinct effectors, at the levels of both signal transduction and chromatin remodeling, in relapsed/refractory AML’s, including *Flt3ITD+ve,* described with a signature of repressed tumor suppressor genes, involving Wnt antagonist *RUNX3*, occurring along with *ID1* and *HOXA* over-expressions. We tracked patient response to combination of Flt3/Raf inhibitor, Sorafenib, and Vorinostat, pan-histone deacetylase inhibitor, without or with added Bortezomib, in consecutive phase I trials. A striking association of rapid objective remissions (near-complete, complete responses) was noted to accompany induced early pharmacodynamic changes within patient blasts in situ, involving these effectors, significantly linking *RUNX3*/Wnt antagonist de-repression (80%) and *ID1* downregulation (85%), to a response, also preceded by profound *HOXA9* repression. Response occurred in context of concurrent *TET2* mutation/hypomorphy and *Flt3ITD+ve* mutation (83% of complete responses). Addition of Bortezomib to the combination was vital to attainment of complete response in *Flt3ITD+ve* cases exhibiting such Wnt pathway dysregulation.

## INTRODUCTION

Novel disease targets are necessary so as to improve acute myeloid leukemia (AML) patient outcomes, and epigenetic pathways are a focus.[1-3] We identified an oncogenic (*Flt3ITD*) pathway to epigenetic repression for the tumor suppressor *DAPK1*, by interaction of p52NF-κB and histone deacetylases (HDACs) on the promoter.[4] Suboptimal efficacy of single Flt3-selective tyrosine kinase inhibitors (TKI) is thought to involve such epigenetic collaboration.[4-6]

Wnt antagonist *RUNX3* is a consequential epigenetically-repressed tumor suppressor gene in AML, especially normal karyotype (+/-*Flt3ITD*).[6-8] By such *RUNX3* repression, its function in interruption of Wnt/β-catenin/TCF4-mediated transcription is prevented,[9] which allows β-catenin to promote leukemia stem cells,[10] by affecting *HOXA10/9/CDX4* transcription.[11] *RUNX3* was a prominent methylation-sensitive repression target of dual *Flt3ITD/TET2* mutations in a murine AML model, and was repressed alongside *BCL11b*, another Wnt-β-catenin antagonist.[6] *ID1* hyperexpression was also prominent in the signature along with *HOXA9*, and Id1 has been demonstrated a Wnt agonist.[6, 12]

Poor-risk AML phenotypes, including *Flt3ITD*+ve, also express transcriptionally-active phospho-c-jun, affecting distinct gene activation.[13-18] C-jun acts with Stat5, in activation of transcriptional targets *PIM1, relB, and MEIS1* (a prognostic gene functioning in leukemic stem cells).[15, 17, 18] Further, c-jun and β-catenin, reciprocally and cooperatively, bind the others promoter consensus, thus conveying cooperative transactivation, involving TCF4 elements such as regulate *HOXA*s.[11, 19, 20]

If not prevented by an epigenetic influence, the AP-1 family of proteins can also block cell cycle and promote senescence and apoptosis through transcriptional up-regulation of both *DAPK1* and *RUNX3*, as well as *p16INK4A/CDKN2A*.[4, 7, 21, 22] Epigenetically repressed *p16INK4A/CDKN2A* promotes leukemia stem cell upregulation,[23] which is observed in normal karyotype *Flt3ITD*+*ve* and complex karyotype AMLs.[23] However, *p16INK4A/CDKN2A* repression is not observed in good-risk [eg. core-binding factor-associated (CBF+*ve*)] AMLs.[23-25]

We considered these tandem repressed tumor suppressor genes as candidates, among a group of prognostic genes, to form a signature for poor-risk AML vulnerable to an epigenetic targeting agent combination. We chose the Flt3/raf-selective tyrosine kinase inhibitor, Sorafenib, along with HDAC inhibitor, Vorinostat, given together as a doublet, or, additionally, in combination with epigenetically-active Bortezomib.[26-28]

We postulated that reversal, early during therapy, of such a blast cell gene expression signature, may predict an emerging remission, and may reflect an epigenetic reprogramming of poor-risk/refractory AML toward enhanced sensitivity, associated with non-expression of *HOXA,* which mimics core-binding factor translocation-associated (CBF+ve) AML.[29]

While demonstrating the safety of two consecutive regimens, we compared the response rates and depth, as well as mechanistic attributes regulating responses. We found mechanistic signatures regulating achievement of remissions were uniform, but that Bortezomib addition had a dramatic impact on depth and rapidity of responses in *Flt3ITD+ve*, where early reversal of gene expression signature was predictive.

## RESULTS

### Response

Patient characteristics are summarized in Supplementary Information: Supplementary Tables 1-2. A total of 15 patients were enrolled in trial 1 with intent to treat (Supplementary Table 1). In the second trial, 18 patients were enrolled (Supplementary Table 2).

With the first cycle of treatment in trial 1 (Sor/Vor), six/fourteen (43%) patients demonstrated partial remission (PR) and 1/14 (7%) achieved a complete remission (CR) (Supplemental Table 1). The patient (#8) with complex karyotype who achieved a CR with one treatment cycle remained disease-free for 5 months (Supplementary Table 1).

Three/six very good partial responders (# 5, 7, 14) and one marrow inevaluable patient (#9) had *Flt3-ITD*, with pre-treatment peripheral absolute blast counts as high as 50,000-85,000/uL, and all demonstrated complete clearance of peripheral blood blasts within 1 week of initiation of treatment (Supplementary Table 1). Following completion of 2 cycles, one responder in this group had reduction of marrow blasts to 10% accomanying a profound reduction of background cellularity.

Responses in the second trial (Sor/Vor/Bor), were generally more rapid, and complete morphologic eradication of blast cells occurred by 1-2 cycles in a significant fraction of patients with *Flt3ITD*, irrespective of the dose level beyond the run-in dosing of cohort 1-where suboptimal Sorafenib was used to ensure safety (Supplementary Table 2). CR rate approximated 59% in that optimal Sorafenib dosing group (Supplementary Table 2).

### Initial gene expression analysis defines repressed tumor suppressor signature in AML blasts

**Figure 1:**
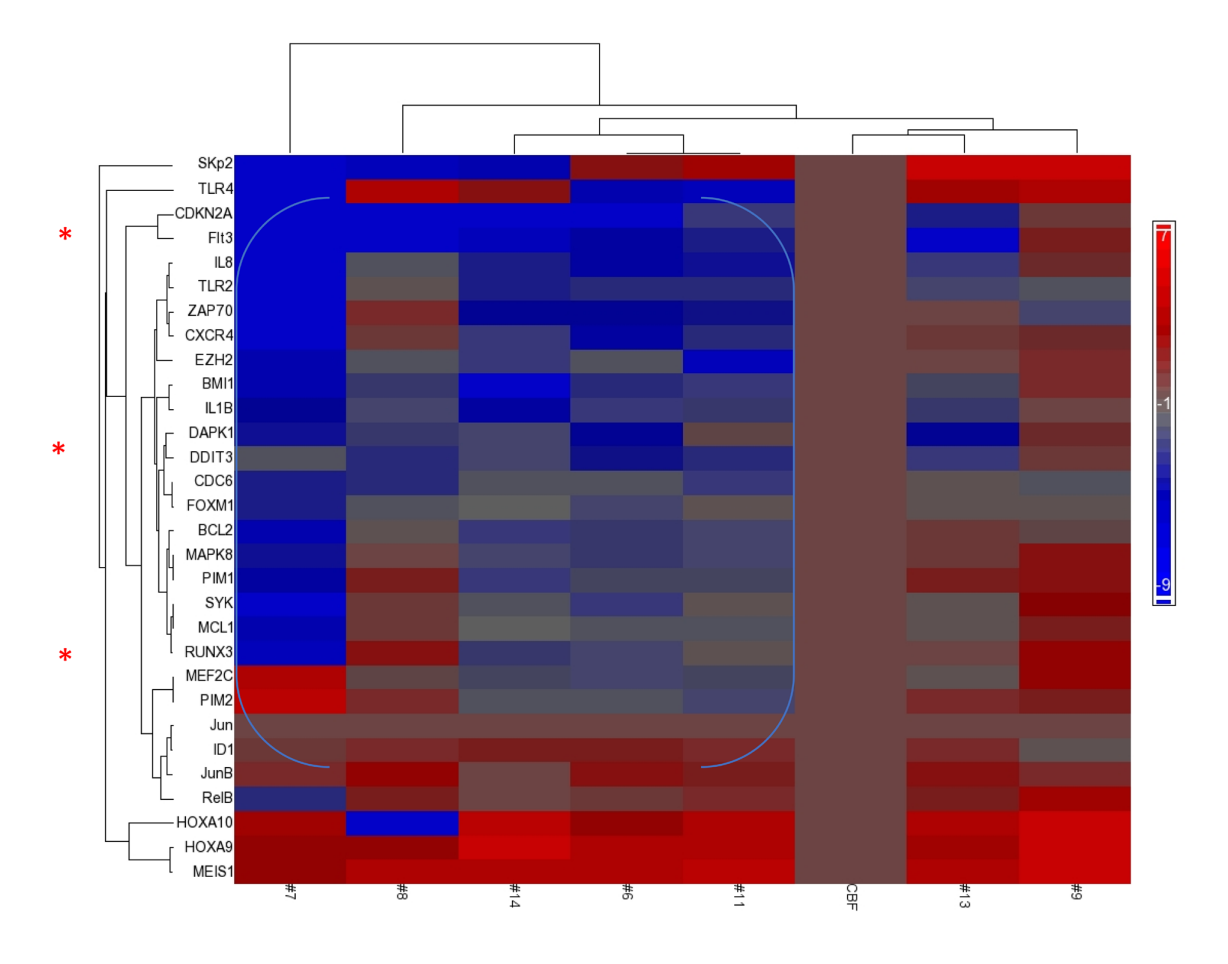
Repression of Tumor Suppressor genes accompanies overexpression of HOXA/MEIS1 Focused GSA (gene set array) expression profile prior to therapy (day 0) of patients treated on first (IUCRO-0234) protocol identifies a group with repression of *DAPK1/CDKN2A/RUNX3* and co-existing *ID1* hyperexpression (note blue bracket) as compared with a control CBF+ve AML with good prognosis. Presented on the horizontal axis, left-to-right, are patients # 7, 8, 14, 6, 11, CBF+ve control, 13, and 9, respectively. Patient #5 had insufficient blast content to perform gene expression analysis prior to therapy, but upon discontinuation of protocol therapy, with disease progression, demonstrated the same phenotype of repression as the bracketed group (data not shown).

Poor prognosis occurs in AML patients whose blasts exhibit repressed expressions of *p16INK4A/CDKN2A* and *DAPK1*, on a background of *HOXA9/MEIS1* over-expression (HS Boswell, unpublished data).[4, 23] These features coincide with *ID1* hyperexpression, which overlaps with these repressed genes, and repressed *RUNX3*.[30] Therefore, we performed focused gene expression analysis on available pretreatment blasts from patients on the first trial, as test cohort (Figure 1).

Compared with the good-risk (CBF+*ve*) gene expression control panel, where *p16INK4A/CDKN2A* is not repressed [23] (Figure 1; data not shown), all these patients’ blasts demonstrated strong over-expression of *HOXA9/MEIS1* on the heat-map, accompanying *CDKN2A* repression. In addition, *ID1* hyper-expression was noted in a subgroup of patients (#6, 7, 8, 11, 13, and 14), where its hyper-expression overlapped with repressed *CDKN2A/DAPK1*, and in most cases, repressed *RUNX3* (Figure 1).

### In trial 1, Sorafenib/Vorinostat promoted changes in Flt3ITD+ve blasts for expression of genes and effector proteins leading to ER stress apoptosis

**Figure 2:**
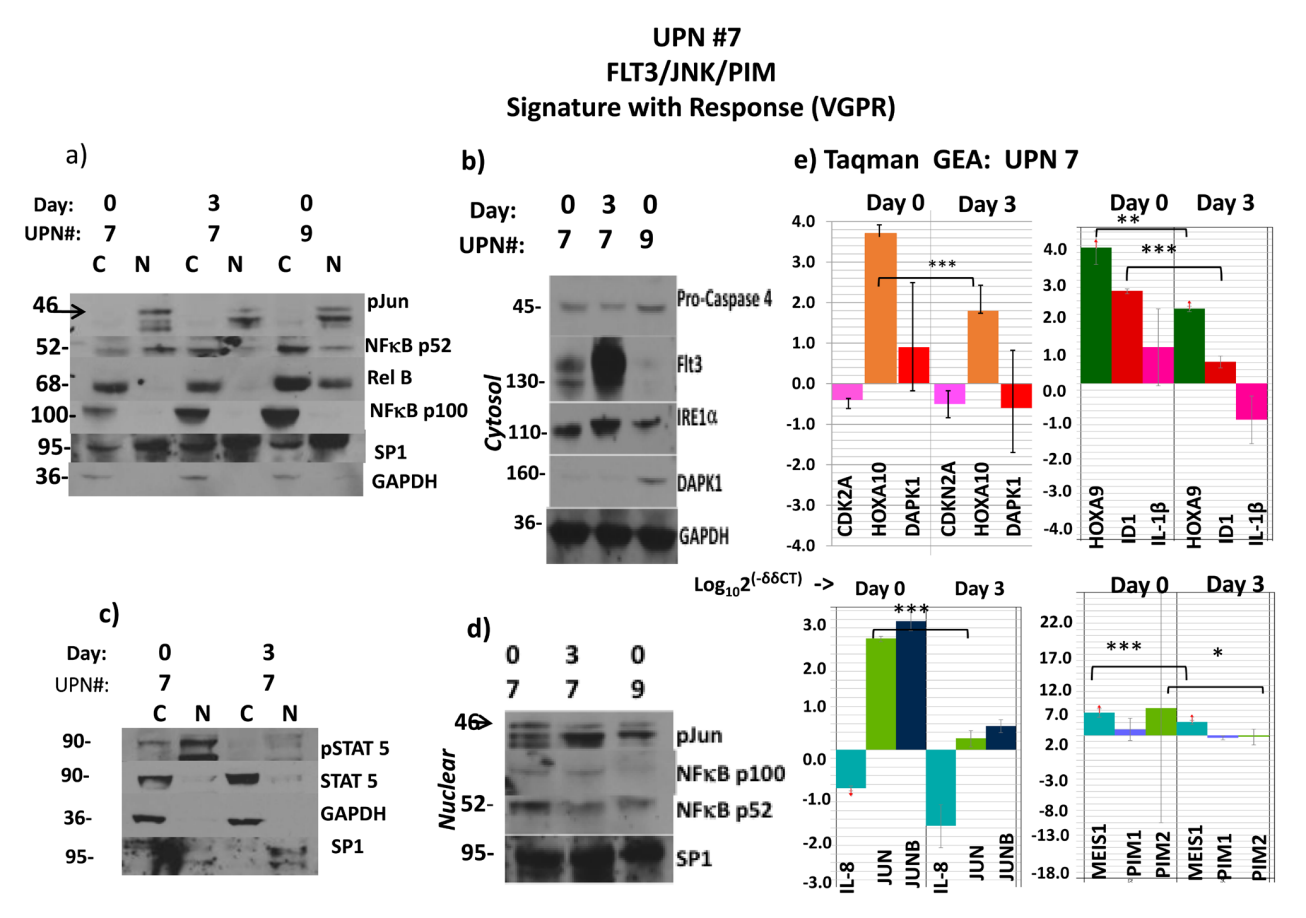
Flt3ITD AML responds to Sor/Vor with strong downmodulation of HOXA’s and MEIS1 accompanying profile of ER stress apoptosis Activity by starting levels: intranuclear p52 NFkB in #7 and #9, 59% and 26%, respectively; phospho-stat5, 78% in #7. These corresponded to higher level of immuno-detected p-jun in #7 and also a lower DAPK1 protein in #7, which would be expected of a cause-and-effect relationship with p52 NFkB- a known *DAPK1* repressor. (**a** to **d**) Sorafenib and vorinostat-induced remission in patient #7 is associated with induction of ER stress apoptosis pathway (Flt3 relocalization, elevated IRE1α, cleavage (70% densitometric) of procaspase 4) despite failure to upregulate low-level expression of DAPK1. However, both p52NFkB and phospho-stat5, which also drive transcriptional expression of *PIM1/2* were severely depleted, leading to an alternative route to ER stress apoptosis. C stands for cytosolic fraction and N stands for nuclear fraction. Molecular weights in the immunoblots are indicated. (**e**) The remission in patient #7 was also associated with severe reduction of *JUN* and *ID1* transcripts, as well as *MEIS1* and *PIM2* transcripts, and significant depletion of *HOXA10/9* transcripts. However, *CDKN2A* transcripts were not augmented in this case. Molecular weights in the immunoblots are indicated.

Because of the slower/lower depth of/ response to the initial regimen (Sor/Vor), an association of changes between gene expression with phosphoprotein signaling intermediates upstream was possible. Immunoblot analysis was performed on pre-treatment blasts with sufficient content: normal karyotype Flt3-ITD+ve patients #7 vs. #9, which accurately demonstrated the heterogeneity expected for activity and subcellular localization of phospho-intermediates based on the gene expression analysis (Figure 2a vs. Figure 1).

Upon initiation of therapy in patient #7 at day #3, where the marrow was still replete with blasts, strong reductions within lysates of nuclear phospho-c-jun (70% reduction by densitometry: Figure 2a and 2d), p52NF-κB (70% reduction: Figure 2a and 2d), and phospho-stat5 (80% reduction: Figure 2c) occurred, and re-localization of stat5 and p52 NF-κB into the inactive cytoplasmic pool was observed (Figure 2a and 2c). However, neither *DAPK1* nor *CDKN2A* mRNAs were de-repressed (Figure 2e); nevertheless, the multi-responsive p52NF-κB/stat5/c-jun-dependent *PIM1* and *PIM2* genes were diminished (p<0.08 and *p<0.05, respectively), and the stat5/c-jun-dependent *ID1* transcripts were reduced by almost 2 logs (Figure 2e, ***p<0.0002). [A day 3 protein analyte was not available from patient #9.] We also observed relocalization of Flt3 to a surface/inactive pool, associated with manifestations expected of ER stress apoptosis upon PIM kinase loss (and 4EBP1 reregulation) in patient #7 lysates[31] (Figure 2b).

The genes whose expression was most significantly decreased in the early-phase of therapy in the marrow blasts of patient #7 were *JUN*, *MEIS1* and *ID1* (***p< 0.00002, ***p<0.00003, and ***p<0.0002, respectively) (Figure 2e). Interestingly, the strong reduction of peripheral blood and bone marrow blasts that followed (Supplementary Table 1) was also preceded by sharp *HOXA9/10* downregulation in day #3 blasts (**p<0.05, ***p<0.005, respectively) (Figure 2e). *RUNX3* (not shown) was not de-repressed in this patient’s blasts, perhaps because c-jun/JUN (a transactivator of *RUNX3*, *DAPK1*, and HOX*A10*) was so strongly expressed initially, and then extinguished by treatment.

**Figure 3:**
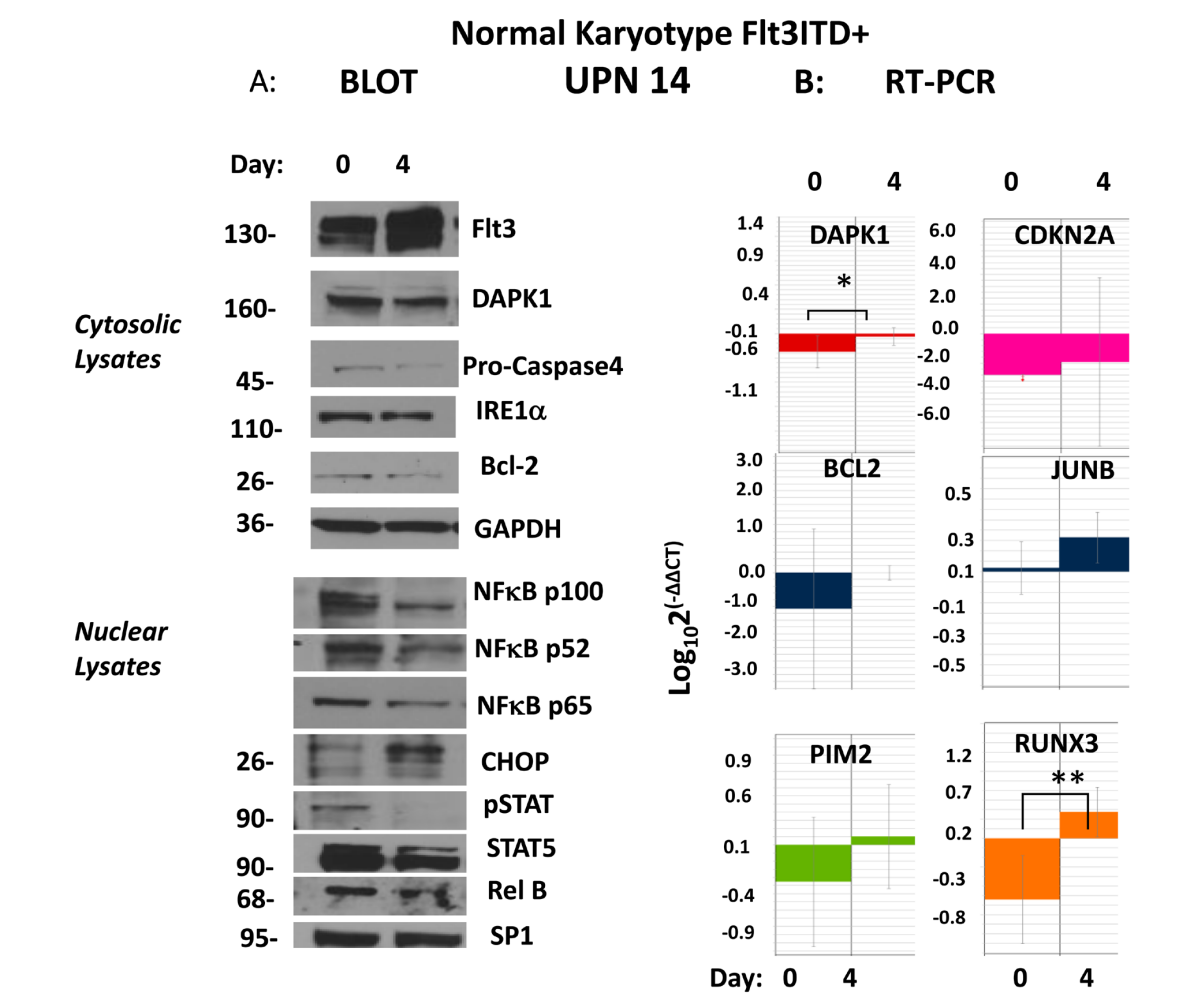
Upregulation of DAPK1/RUNX3/CDKN2A presages remission following evolution of ER stress apoptosis (**a**) A patient with normal karyotype, Flt3ITD (#14) at pretreatment evaluation demonstrated by immunoblot strong activation of phospho-active stat5 and p52NFkB as well as having strong repression (compared to CBF+ve control AML) *CDKN2A*, and *DAPK1*, as well as lesser repression of *RUNX3*. Early evidence for evolving remission after 3 days demonstrated by immunoblot involved strong upregulation of CHOP, cleavage of caspase 4 (80% densitometric) evidencing ER stress apoptosis occuring in context of strong depletion of stat5/p52NFkB activation. Molecular weights in the immunoblots are indicated. (**b**) In addition, upregulation of transcripts for *DAPK1, CDKN2A and RUNX3* were demonstrated.

Normal karyotype *Flt3ITD+ve* patient #14, whose blast gene expression heat-map similarly revealed the triple-repressed tumor suppressor gene signature noted above (Figure 1), was pharmacodynamically monitored with therapy, which demonstrated a different mechanistic complement and a distinct route to an ER stress apoptotic outcome in blasts, independent of PIMs (Figure 3). A dramatic response to Sor/Vor therapy ensued (Supplementary Table 1). Pre-treatment marrow blasts demonstrated significant, but more limiting, intranuclear levels (vs. #7 (Figure 2)) of phospho-stat5 and p52NF-κB (Figure 3a, left panel). At treatment day #4, nuclear lysates of blasts revealed total loss of p-stat5, and significant reduction of p52NF-κB (65% reduction by densitometry). This occurred in the setting of 5-fold up-regulation of *DAPK1* transcripts by real-time qRT-PCR (*p<0.04) and stable DAPK1 protein levels, along with 3-fold up-regulation of CHOP (Figure 3a, left panel). Expected ER stress apoptosis evolution in blasts was further evidenced by Caspase 4 cleavage (>80% by densitometry) (Figure 3a, right panel). Indeed, this apoptotic process occurred in the absence of downregulation for *PIMs*, whose starting expression was quite low in context of low starting levels of p-jun and p52NF-κB vs. patient #7 (Figure 2 and data not shown).

In addition, the starting expression of *HOXA’s* was much lower here, in keeping with modest levels of *JUN* (<200-fold less *JUN* than patient #7) as driver, affecting an absent p-jun band visible by immunoblot (data not shown). The 8-fold up-regulation of *RUNX3* transcripts observed by real-time qRT-PCR (**p<0.006) (Figure 3b) was not associated with any change in putative target. There was derepression in *CDKN2A* transcripts of borderline significance (p=0.07), and no change in *ID1* expression (Figure 3b; and data not shown).

Thus, the mechanism to effect apoptosis in these *Flt3ITD+* blasts was distinct from those *Flt3ITD+* blasts patient #7, and the de-repression of *DAPK1* observed here appears to result from the inhibition of the repressive influence of p52NF-κB with HDACs.[4]

On the other hand, patient #6 without *Flt3ITD*, enrolled into dose-level 2 with optimal Sorafenib dosing and proven Vorinostat pharmacodynamics,[32] did not respond (Supplementary Table 1). We found failure to reduce baseline nuclear p52NF-κB levels, rising p65NF-κB levels, and rising, extremely high HOX*A9/MEIS1* at day 4 (data not shown), supporting the importance of the predictive findings.

Evidence for activity of the Sor/Vor drug combination was also present among cases with complex/poor-risk cytogenetics (Supplementary Table 1), where *CDKN2A* repression is well described.[23, 33] Pretreatment blasts patient #8 demonstrated strong *CDKN2A* repression and *ID1* hyperexpression (Figure 1), and early with therapy an early 3-fold log-reduction of *ID1* transcripts (**p<0.01) and an elevation of *CDKN2A* transcripts of at least 2-logarithms (***p<0.003) occurred (data not shown).

### Dramatic increase occurs in depth and rapidity of responses in *Flt3ITD+ve* disease by addition of Bortezomib, involving *ID1/RUNX3/HOXA* axis.

In the second trial including Bortezomib, responses were seen beginning at cohort 2 (which involved resumption of Sorafenib at efficacious single-agent dosing and schedule) (Supplementary Table 2). These responses demonstrated no association to level of Bortezmib escalation in dose or schedule. Nor did responses relate to resumption of the full schedule of Vorinostat, as achieved in the prior protocol (Supplementary Tables 1, 2). However, responses were limited to *Flt3ITD+ve* patients (Supplementary Table 2).

**Figure 4:**
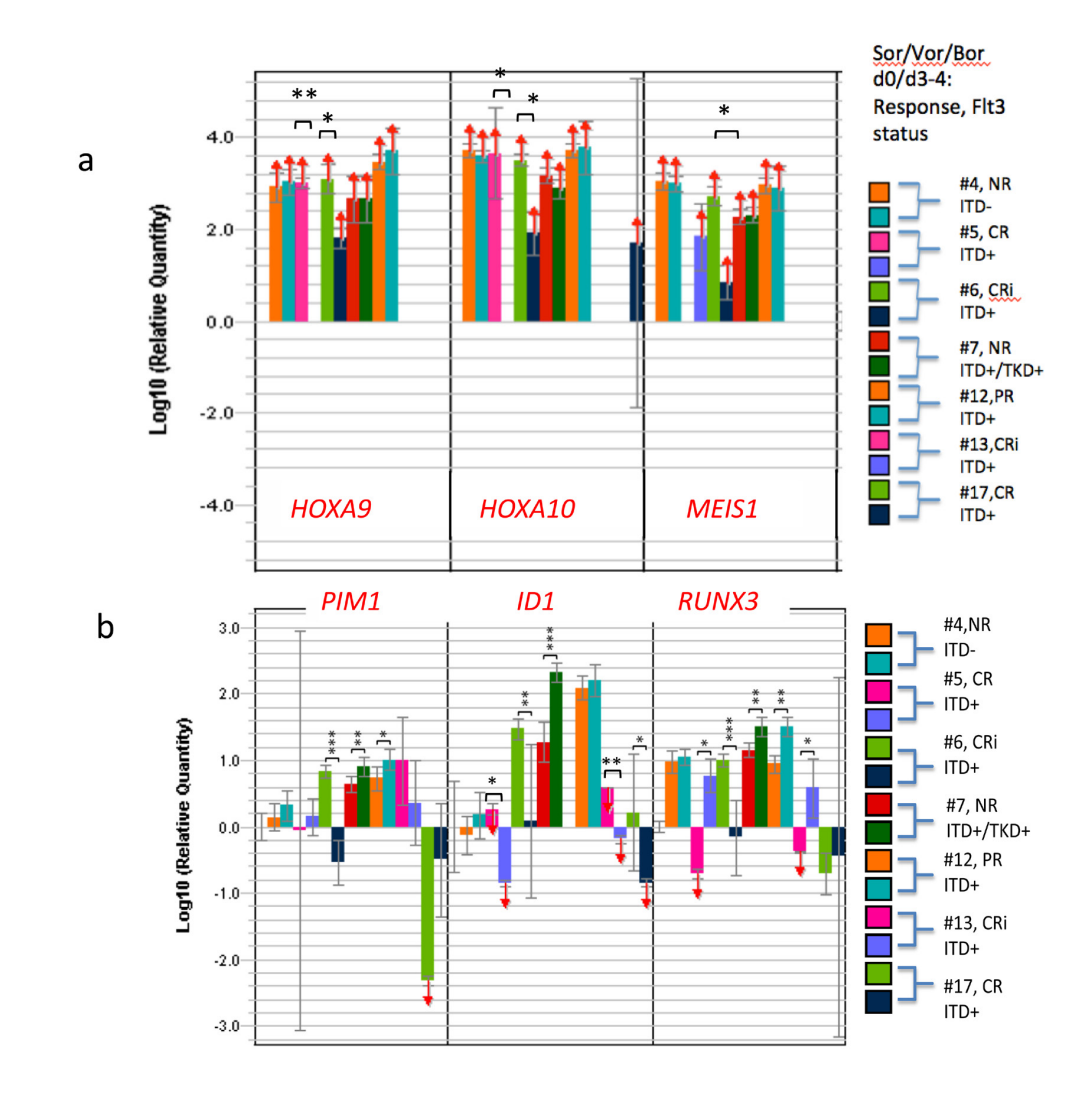
Coupling of RUNX3 upregulation and/or ID1 downregulation along with HOXA reductions early after Sor/Vor/Bor leading to remissions Transcriptional changes induced by combination of Sor/Vor/Bor regimen in patients, comparing pretreatment marrow blasts with day3/4. Patient #5 had total reduction of *ID1* transcripts and significant upregulation of *RUNX3* following initiation of therapy. The same outcome was seen in patient #13. Patient #6 had severe reduction of *PIM1* as well as *ID1* transcripts upon initiation of therapy. The most common result for responses was severe depletion of *ID1* and upregulation of *RUNX3*, associated with *HOXA9/10* depletion. *TET2* transcript hypomorphy was strongly associated with these outcomes (Supplementary Table 3).

Eradication of peripheral blasts was hastened, and marrow cellularity at day 4 was too limiting to allow immunoblotting analyses. However, gene expression from marrow blasts (day0/4) was quite informative, and relayed the previous trends, particularly in the *HOXA/RUNX3/ID1* axis (Figure 4).

Among patients in cohort 2 (dose-level 1) (Supplementary Table 2), strong *HOXA9* and *HOXA10* expressions in the pretreatment blasts were sharply log-reduced or extinguished early into therapy at day 4, when a subsequent CRi later evolved (*HOXA9*: patient #5, **p<0.01; patient #6, *p<0.05; *HOXA10*: pt#5, *p<0.05, pt#6, **p<0.01) (Figure 4). On the other hand, no such reductions occurred in non-responding patients, or in patient #12 with a PR achieved with the 1^st^ cycle that did not improve with cycle #2 (Figure 4a). In the CRi responders (#13 and #17) in cohorts 4 and 5, *HOXA* expression and *MEIS1* expression were limiting to begin with (Figure 4a), perhaps related to the much lower c-jun activity capable of driving *HOXAs/MEIS1* than in the aforementioned cases, similar to the situation that obtained with patient #14 in trial 1 (Supplementary Table 1, Figure 3).

In the CRi responders, response was heralded in blasts by early sharp downregulation of *ID1* (pt #5, ***p<0.005; pt #6,**p<0.005; pt# 13, **p<0.005, pt# 17, *p<0.05) (Figure 4b). In all of the blasts of CR/CRi responders, including the CR from the first trial (5/5), *ID1* was sharply downregulated early by therapy (Figure 4b and Supplementary Table 3) (p<0.002 compared with PR/nonresponders). In all but two of patients with CR/CRi/VGPR achieved across both trials, early *RUNX3* upregulation in blasts (5/7) was associated with that response (Figure 4b, Figures 2-4, Supplementary Table 3), and in those outlier cases, sharp downregulation of *JUN*/cjun, a HOX/MEIS1 transactivator, occurred as an alternate route to the endpoint (#7, trial 1, #6, trial 2[data not shown]). In the latter example (trial 2, patient #6), absent *RUNX3* upregulation, *PIM1* downregulation as well as *ID1* downregulation was noted in association with response (Figure 4). None of the patients’ blasts attaining CRi in the second trial demonstrated significant *CDKN2A* upregulation, whereas both the VGPR#14 and CR#8 in trial #1, upregulation of *CDKN2A* occurred (Supplementary Table 3).

Induced *ID1* hypo-expression occurred in 4/4 CR/CRi’s and 5/6 VGPR/CR/CRi and was associated with both *RUNX3* de-repression/upregulation, *HOXA* diminution and *CDKN2A* de-repression. However, absent VGPR/CR/CRi, *ID1* depletion did not occur (p<0.002).

### Vulnerability of *Flt3ITD+ve* AML with *TET2* loss and heightened *HOXA’s*

Haploinsufficient mutations of *TET2* gene, as well as miRNAs targeting 5’-3’ destruction of its mRNA, affect epigenetic functions.[34-36] In either case, functional transcript reduction ≥35% yields a hypomorphic phenotype; affecting repression of Wnt pathway antagonists, which are WT1 target genes. [1, 6, 13, 25, 37, 38] Poor clinical outcomes result.[39]

**Figure 5:**
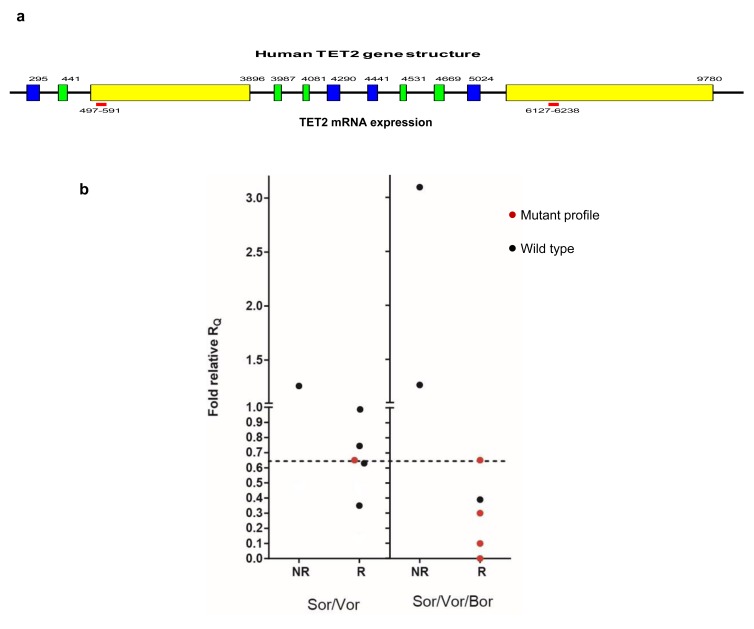
Mutant TET2 marks response to Sor/Vor or Sor/Vor/Bor *TET2* transcript relative quantity was plotted in comparison to CBF+ve control for the two trials, distinguishing responders vs. nonresponders. Mutant transcript profile was defined as quantity of 3’/5’ transcript quantity equal to or less than 0.65x control, whereas non-mutant transcript quantities were defined based on conventional detection methods.

We postulated poor outcome might be linked to *RUNX3* repression, in part, through heightened *HOXA* signaling (as consequence of unrestrained β-catenin[9]). Available blast cells from our two trials were analyzed for existence of truncated/mutant *TET2* mRNAs (failed elongation/detection occurs with common nonsense/frameshift mutation) as well as reduction of complete transcripts by antagonistic miRNA’s (Figure 5, Supplementary Table 3). We stratified samples in comparison to the same CBF+ve controls as before (because *TET2* mutation is not associated with inv(16) CBF AML:(4) TCGA database <2%), and placed a distinction between samples with more or less than 65% of the high-expressor controls for *TET2* transcripts to distinguish a hypomorphic phenotype.

*TET2* transcript deficiency of starting blasts signaled enhanced activity of the combinations, especially Sor/Vor/Bor (Figure 5). This was noted by severe reduction (or ablation) of the 3’/5’ transcript ratio (corresponding mutation) in the pretreatment blasts of majority responders, especially in the second trial (Figure 5). Proper assignment was confirmed with independent primers in the TaqMan plate (data not shown). In addition, preexisting blast *TET2* hypomorphy most commonly associated with *RUNX3* derepression upon initiation of therapy (Supplementary Table 3). Among 15 samples across both trials available for multigene molecular analysis, early relief of *RUNX3* repression occurred in 8/10 responders (5/7 CR/CRi/VGPR), whereas 2/3 non-responders with samples available for analysis failed to demonstrate *RUNX3* de-repression (p=0.03, Chi square).

*RUNX3* de-repression and/or *JUN*/c-jun downregulation was linked to early treatment-induced diminution of *HOXA9/10* expression in blasts and subsequent hematologic response (Supplementary Table 3). As noted above, this was also associated with early *ID1* hypo-expression, while induced along with *HOXA* diminution. Thus, a pathway of epigenetic repression was alleviated by the combination therapy involving signature *ID1-TET2-RUNX3*, and *HOXA* depletion was linked to modulation of this pathway and *JUN*/c-jun.

## DISCUSSION

AML relapse/resistance after therapy may be epigenetically determined. Mutant epigenetic modifiers that may persist in remission and contribute to relapse are *mDNMT3A, mASXL1, mTET2*. As a consequence, at least in the first two genes, hypomethylation on *HOXA* alleles, and resultant *HOXA* hyperexpression occurs.[40-42] By contrast, good risk CBF *+ve* AMLs, which display highly durable induction/consolidation chemotherapy outcomes, display severe *HOXA/B* hypoexpression within bulk and CD34+ cells, in association with leukemic stem cell content of limited depth.[24, 29] Thus, modulation of chemosensitivity may require diminution of the chemoresistance effector and LSC driving factor *HOXA9*. [11, 24, 43]

Here, we investigated the impact of *TET2* hypomorphy on a putative pathway to affect unrestrained *HOXA* overexpression, which may be a consequence of repression in RUNX3- an inhibitor of *HOXA* driver β-catenin. We postulated AMLs with this signature might be affected by an epigenetically-active targeting combination given to patients. In addition, we postulated recognized positive regulators of the pathway to *HOXAs: c-jun* and *Id1,* may be sensitive to the combination.[11, 19, 20]

Combined administration of Sorafenib and Vorinostat, especially along with Bortezomib, induced rapid and deep remissions in patients *Flt3ITD+ve*, commonly characterized by early limitation of blast *HOXA’s* expression. Such induced remissions were accompanied in 80% cases with upregulation of repressed *RUNX3*, and were also strictly linked to *ID1* downregulation. Reversal of the epigenetic signature by the targeting combination appears to signal mutual involvement of members in leukemic genesis as observed in a mouse model,[6] and signature reversal was a descriptor of subsequent remission. However, because RNAseq and/or protein interaction studies were not performed here, conclusive assignment of a direct converging pathway cannot be made.

Introduction of Bortezomib within the regimen sharply increased rapidity and depth of response to the combination in *Flt3ITD+ve* cases, and Bortezomib has demonstrated effect on transcriptional and posttranscriptional inactivation of Flt3.[26-28] Bortezomib was also found by others to downregulate *HOXA9* in human *Flt3ITD* AML cells, while sensitizing the cells to chemotherapies and TKIs, which involved an induced repression of *HOXA9,* by rescue from proteasomal destruction of histone H3 K27 methylator- EZH2, the enzymatic effector of polycomb repressive complex 2.[43] In the *Flt3ITD+ve* cohort treated with the Bortezomib combination, there were insufficient blast cells at day ¾ to address these issues fully, as a direct result of the activity of the combination. However, we previously tested Bortezomib with and without TKI *in vitro* on other genotypically similar *Flt3ITD+ve* AMLs, and demonstrated sharp Flt3 protein loss within cell lysates with short treatment. On the other hand, in our trial we did analyze *EZH2* mRNA before and at day ¾ of treatment in situ without a clear predictive level of *EZH2* transcripts at these timepoints for determining subsequent response.

Thus, Sor/Vor/Bor is a novel combination with promise for priming poor-risk AMLs with this gene expression signature, particularly *Flt3ITD+ve* with *TET2* mutation, for subsequent therapeutic sensitization (Supplementary Table 3). Indeed, the capacity for this regimen to be a bridge-to-transplant with prolonged subsequent disease-free survival was demonstrated, and additionally another patient with reversal of this gene signature anticipating the induced remission, had a 5-month unmaintained remission.

We found no evidence for an influence on responsiveness for one accompanying gene mutation frequently occurring with *Flt3ITD:* mutant *NPM1* (data not shown). Recently*,* patient blasts, documented by NGS as bearing complements of *Flt3ITD* with *IDH2/WT1/TET2/DNMT3A* mutations (when alone or in mutually nonexclusive pairs), were also tested *in vitro* for proliferative silencing /apoptosis by Bortezomib with Flt3 inhibitor, and we found efficacy and synergy of the combination in these mutational binary- or ternary-defined subgroups in Flt3ITD*+ve* AML (HS Boswell, in preparation). In fact, because of the strict association at diagnosis, and stability upon relapse, of essentially all Flt3mutant AML’s with accompanying epigenetic mutations within *IDH2/TET2/WT1* pathway or, additionally, with DNMT3A or ASXL1 mutations, this regimen, or a very similar one, may have broad applicability for sensitivity “priming” [43,44].

Our study performed in-depth examination of the early changed protein/gene signature achieved *in situ* within blast cells toward crucial downstream effectors, which revealed significant differences from the mechanism anticipated from Flt3-selective TKI alone. [4, 45, 46] Our data demonstrated, for at least *Flt3ITD+ve/TET2mut/*hypomorphic AML, a more complex and cooperative remission-inducing mitigation, by epigenetic combinations causing de-repression/upregulation of Wnt antagonist *RUNX3*, and affecting severe *ID1* and *JUN*/*c-jun* depletion, which were associated with *HOXA* depletion.[6, 9, 12, 20] By contrast, mechanistic linkage to *CDKN2A/DAPK1* de-repression was infrequent.

## MATERIALS AND METHODS

### Patient selection

In the first trial, all adult patients with a diagnosis of relapsed or refractory AML, and those older than 70 with newly diagnosed disease, were eligible upon informed consent (Supplemental Information), adequate performance status and hepatic/renal function. In the second trial (Supp. Inf.), patient selection was restricted to Flt3ITD+ve, or complex karyotype or monosomy 5/7, from relapsed/refractory patients across the age spectrum, or additionally, from newly diagnosed elderly. All patients had a baseline bone marrow aspiration for staging/diagnosis and pharmacodynamic studies (baseline) as well as cytogenetics and Flt3 mutation analysis.

### Trial designs and treatment

A conventional 3+3 dose-escalation schedule was followed with 3-6 patients per cohort. The dose-escalation schemes for trial 1 are noted under Supplementary Table 1. Sorafenib was given orally at a dose of 400 mg twice daily, and oral vorinostat was given with dose escalation in successive cohorts. Each cycle consisted of 14 days of treatment followed by 7 days of break, 21 days total. A bone marrow exam for response evaluation was performed on day 15 of each cycle. Response was assessed as reported by Cheson.[47] Patients who achieved at least 50% reduction of bone marrow blasts to 5-25%, but not a complete morphologic remission, were eligible to receive the second cycle. Patients, who achieved at least 10% reduction of bone marrow blasts, but not a complete remission with the second cycle, were eligible for a third cycle. Patients received a maximum of 3 cycles in this first trial. No further treatment would be given beyond a complete remission (CR). The dose-limiting toxicity (DLT) was defined as any grade 3 or 4 non-hematologic toxicity using the National Cancer Institute NCI’s Common Terminology Criteria for Adverse Events (CTCAEv3.0).

In the second trial a similar 3+3 design was used (Supplementary Table 2), but the initial schedule involved dose-reduction of Sorafenib to 200mg po bid to avoid possible toxicity associated with addition of a third drug-Bortezomib. This dose level assigned within cohort 1 was deemed run-in (-1) dosing. Also, dosing with Vorinostat was initially reduced in schedule, but not in dose, from optimal schedule in protocol I, to days 1-4, and 8-12, to avoid potential toxicities associated with initiation of the 3^rd^ drug-Bortezomib. No such toxicities were observed, and a 5^th^ dosing level including Sorafenib and Vorinostat, at the full schedule from the first trial, were still compatible with the optimal dose-schedule of Bortezomib (Supplementary Table 2).

### Cells

Bone marrow blast cells were obtained from patients within 7 days prior to treatment and at the end of 3-4 days of therapy, and processed as previously described.[4]

### Real-time RT PCR analysis in a focused gene-set array panel

This was as described.[4] qRT-PCR for 30 AML related genes and one housekeeping gene were performed at low-density array (LDA) format according to the manufacturer’s protocol (TaqMan Gene Expression Micro Fluidic card, 4346799, Applied Biosystems/Life Technologies). 18S rRNA was chosen from TaqMan Human Endogenous Control Plate (Applied Biosystems) as internal control. Relative expression was calculated using RQ manager Ver 1.2 (Applied Biosystems) using one-patient volunteer as a calibrator sample (fusion core-binding factor-positive (CBF+*ve*[inv(16)]), negative for *Flt3ITD*, and very low *c-jun* and *Meis-1* expression). In addition, the CBF*+ve* control had high expression of *TET2* mRNA, as expected based on the rarity of *TET2* mutation within this good-risk AML subgroup (see below). Copy number or fold-change in expression was calculated using the 2-∆∆Ct method.[48] For initial definition of a tumor suppressor gene “repression cohort” among the group of patients accrued to the trial with intention-to-treat, baseline expression data of 29 test genes was normalized to expression of c-jun, which is a known trans-activator of the core tumor suppressor target gene cohort (*CDKN2A, DAPK1, RUNX3)*, and *MEIS1*.[4] Data were conveyed into heat-map format reporting ∆CT values and statistical analysis was applied to these values, using 18S RNA as control value.

### Real-time RT-PCR analysis of *TET2* expression

Two real-time PCR primer pairs were designed to amplify the 5’ (497-591bp) and 3’ (6127-6238bp) part of human *TET2* mRNA respectively. The sequences are: *TET2*-5’ forward: 5-GATAGAACCAACCATGTTGAGGG, *TET2*-5’ reverse: 5-TGGAGCTTTGTAGCCAGAGGT, *TET2*-3’ forward: 5-GCATGCCACAACCCCTTTAA and *TET2*-3’ reverse: 5-CCAAAGAGCCAAGCCATGTT. Expression of the 5’ and 3’ part of TET2 mRNA was first normalized by GAPDH expression and then the expression ratio of 3’ mRNA to 5’ mRNA was calculated using 2^-ddCt^ method. GAPDH primer sequences are: forward: 5-GACCACAGTCCATGCCATCACT and reverse: 5-GCTTCACCACCTTCTTGATGTCA. Data for transcripts detected with an updated microfluidic card including *TET2* were obtained in the second cohort, and analyzed by the aforementioned protocol.

### Immunoblot analysis

Cytosolic or nuclear proteins were subjected to Western blotting with indicated antibodies as described.[4]

### Statistical analysis

Individual patient gene expression data within the defined cohorts was subjected to population analysis for statistical distinction using Mann- Whitney/Wilcox methodology and reported as *p* value for null hypothesis at 95% confidence interval. Additionally, t-test methodology was also used for comparison of individual patient samples for distinction of gene expression change upon patient treatment.

## SUPPLEMENTARY MATERIALS TABLES


