# Supplementary material for: Consecutive epigenetically-active agent combinations act in ID1-RUNX3-TET2 and HOXA pathways for Flt3ITD+ve AML
Source: Oncotarget. 2017 Dec 25;9(5):5703–15. doi: 10.18632/oncotarget.23655 (PMC5814168; doi:10.18632/oncotarget.23655)
Supplement: Supplementary file 1 [file oncotarget-09-5703-s001.pdf]

Consecutive epigenetically-active agent combinations act in *ID1-RUNX3-TET2* and *HOXA* pathways for *Flt3ITD+ve* AML

SUPPLEMENTARY MATERIALS

Clinical trials

The clinical trials were approved by the Indiana University Institutional Review Board. Clinical outcomes analysis was performed by Drs. Sayar, Abu Zaid, and Boswell, and the information was available to all the collaborators.

Protocol synopses

Patients with relapsed/refractory AML, or primary diagnoses occurring in elderly patients (=/>70 years), were treated on consecutive phase I studies, following informed consent according to standard guidelines of the Helsinki Declaration.

Phase I, Open-label, Dose-escalation Study of the Combination of Sorafenib and Vorinostat in Poor-risk Acute Myelogenous Leukemia (AML) and High Risk Myelodysplastic Syndrome (MDS)

IUCRO-234 (ClinicalTrials.gov Identifier: NCT00875745)

Primary outcome measures:

Determine the maximum tolerated dose of a combination of Sorafenib and Vorinostat administered to patients with poor-risk AML, or MDS with >10% blasts: Baseline through cycle 3

Secondary outcome measures:

Evaluate response and the duration of response to this combination targeted therapy

Evaluate the toxicity of the combination

Exploratory objective

To explore the mechanistic attributes for efficacy of this combination in AML with respect to expression of a panel of prognostic genes including c-jun, stat5, and NFkB

2. Phase I/II Study of Combination of Sorafenib, Vorinostat, and Bortezomib for the Treatment of Acute Myeloid Leukemia With Complex- or Poor-risk (Monosomy 5/7) Cytogenetics or FLT3-ITD Positive Genotype

IUCRO-0327 (ClinicalTrials.gov Identifier: NCT01534260)

Primary outcome measures:

Number of dose limiting toxicities (DLTs) after administration of sorafenib, vorinostat and bortezomib

Secondary outcome measures:

Number of patients with a partial response or greater to the combination of sorafenib, vorinostat and bortezomib of treatment]

Duration to relapse in patients who experience relapse following achievement of a complete remission. This is defined as the duration of remission from the time of documentation of complete morphologic remission to the time of documentation of relapse.

Exploratory objective:

To explore the mechanistic attributes for efficacy of this combination in AML with complex- or poor-risk (monosomy 5/7)- cytogenetics or FLT3-ITD positive genotype.

Outcomes. Toxicity was recorded for the two trials as noted above.

In the first trial, a total of 15 patients were enrolled with intent to treat, and 3 dose levels were assigned. 13 patients completed at least 1 cycle of therapy (one patient withdrew and another failed to complete). The 3 patient cohorts were treated as following: Sorafenib 400 mg twice daily in all cohorts.

Vorinostat – 100 mg twice daily in cohort 1;
200 mg in AM and 100 mg in PM in cohort 2;
200 mg twice daily in cohort 3.

No DLT was observed in the first two cohorts. One/six patient in the 3rd cohort developed grade 4 diarrhea in the setting of neutropenic fever. A maximum tolerated dose (MTD) was not achieved in the study, but an optimal dose was determined as that dosing achieved in cohort 3.

In the second trial, which involved integration of Bortezomib into the first trial's optimal dosing for Sorafenib and Vorinostat, required initial recission of optimal dosing achieved above, followed by stepwise escalation to avoid DLTs:

Dosing levels:

-1 (this is run-in dosing below Sorafenib efficacy level to determine safety): Sorafenib 200mg bid, days 1-14, Vorinostat 200mg bid days 1-4, and 8-12, Bortezomib 1.0mg/m2 days 1, 8 of a twenty-one day cycle

1: Sorafenib 400mg bid, days 1-14, Vorinostat 200mg bid days 1-4, and 8-12, Bortezomib 1.0mg/m2 days 1, 8 of a twenty-one day cycle

2: Sorafenib 400mg bid, days 1-14, Vorinostat 200mg bid days 1-4, and 8-12, Bortezomib 1.3mg/m2 days 1, 8 of a twenty-one day cycle

3: Sorafenib 400mg bid, days 1-14, Vorinostat 200mg bid days 1-4, and 8-12, Bortezomib 1.3mg/m2 days 1, 4, 8, 11 of a twenty-one day cycle

4: Sorafenib 400mg bid, days 1-14, Vorinostat 200mg bid days 1-14, Bortezomib 1.3mg/m2 days 1, 4, 8, 11 of a twenty-one day cycle

18 patients were enrolled in the phase I portion.

The response/efficacy measures of the two trials are reported, respectively, in Supplementary Tables 1 and 2

Supplemental Table 1: Patient characteristics for Sorafenib/Vorinostat trial

Cohort	Patient	Age/Sex	AML status	Cytogenetics	FLT3 -ITD	Response	Toxicity	Baseline/ Postcycle 1 BMBP	Baseline/ Postcycle 1 PBABC
1[a]	1	71/M	3rd rel	Normal	-	No	None	50/68	0.9/2.3
	2	56/M	Rel, post transplant	Normal	-	No	G1 Hand-foot	57/70	47/38
	3	65/M	Primary ref	-7	-	PR	None	27/10	0/0
2[a]	4	72/M	3rd rel	t(1;6)	-	No	None	80/70	0.9/1.5
	5	72/M	Rel/ref	t(18;21)	+	PR	None	24/12	1.3/0.5
	6	37/F	Rel/ref	Normal	-	No	G2 nausea	89/76	2.9/11.7
3[a]	7	58/M	Rel/ref	Normal	+	PR	None	79/25[b]	31.8/0
	8	74/M	New	-5, -7	-	CR	None	67/3	0.5/0
	9	59/F	Rel/ref	Normal	+	NE	G3/4 diarrhea	80/NE	6.3/NE
	10	46/F	Rel/ref	Complex	-	No	None	34/86	0/0.5
	11	74/M	Primary ref	Complex	-	PR	None	50/25	12.9/0.5
	12	60/F	Primary ref	Normal	-	No	None	53/47	0.2/0.2
	13	70/F	New	Normal	-	PR	None	56/15	0/0
	14	42/F	Primary ref	Normal	+	PR	None	89/25[c]	80/0

Abbreviations**:**

BMBP – Bone marrow blast percentage, CR – Complete remission, G – grade, PBABC – Peripheral blood absolute blast count (k/ml^3), NE – Not evaluable, PR – Partial remission, Ref – Refractory, Rel – Relapsed

a= Sorafenib 400 mg twice daily in all cohorts.

Vorinostat – 100 mg twice daily in cohort 1; 200 mg in AM and 100 mg in PM in cohort 2; 200 mg twice daily in cohort 3.

b= 15% blasts after 2 cycles

c= 10% blasts on 10-20% cellular background after 2 cycles

**Note: patient 15 was enrolled but withdrew prior to completing treatment. At that time, there was evidence for progression in the peripheral blood.

Supplemental Table 2: Patient characteristics for Sorafenib/Vorinostat/Bortezomib trial

Dosing level	Cohort	Patient	Age/ Sex	AML status	Cytogenetics	FLT3-ITD	Response	BMBP Baseline/Post subsequent cycles	PBABC Baseline/Post subsequent cycles
-1	1	1	33/M	Relapsed post-allo	-7	-	Not evaluable	76	8.8
		2	67/M	Re/Re post-allo	-7q	+	Not evaluable	64	7.2
		3	64/F	Refractory	Complex	-	No response		
		4	54/F	Relapsed	Complex	-	No response	97	
1	2	5	55/F	Relapsed	Normal	+	CRi	55/2	0
		6	67/M	Relapsed	Normal	+	CRi	90/25/<5	20/0
		7	64/M	Refractory	Normal	+	Initial response	87/10/77	2.8/0/0.4
2	3	8	35/M	Refractory	Unavailable	+	Not evaluable	95	12.6
		9	73/M	Untreated	Complex	-	No response	36	0
		10	51/M	Relapsed	Complex	-	No response	20/25/30	0
		11	63/M	Refractory	Complex	-	No response	65/65	0.7/0.8
3	4	12	46/F	Refractory	46,XX,t(2;8)	+	Initial response	86/48/68	11.6/0/0.4
		13	50/F	Refractory	Normal	+	CRi	66/33/10/2	1.5/0/0/0
		14	37/M	Relapsed post-allo	Unavailable	+	CRi	73/26/1	0.5/0
4	5	15	40/F	Refractory	Unavailable	+	No response	33/56	0.1/0.2
		16	46/M	Refractory	-7/Complex	-	No response	20/20	0.1/0.2
		17	24/F	Relapsed post-allo	46,XX,t(12;15)	+	CRi	83/8/2	3.1/0.1/0
		18	26/M	Refractory	Normal	+	CRi	24/3	0/0

Dosing levels:

-1 (this is run-in dosing below Sorafenib efficacy level to determine safety): Sorafenib 200mg bid, days 1-14, Vorinostat 200mg bid days 1-4, and 8-12, Bortezomib 1.0mg/m2 days 1, 8 of a twenty-one day cycle

1: Sorafenib 400mg bid, days 1-14, Vorinostat 200mg bid days 1-4, and 8-12, Bortezomib 1.0mg/m2 days 1, 8 of a twenty-one day cycle

2: Sorafenib 400mg bid, days 1-14, Vorinostat 200mg bid days 1-4, and 8-12, Bortezomib 1.3mg/m2 days 1, 8 of a twenty-one day cycle

3: Sorafenib 400mg bid, days 1-14, Vorinostat 200mg bid days 1-4, and 8-12, Bortezomib 1.3mg/m2 days 1, 4, 8, 11 of a twenty-one day cycle

4: Sorafenib 400mg bid, days 1-14, Vorinostat 200mg bid days 1-14, and Bortezomib 1.3mg/m2 days 1, 4, 8, 11 of a twenty-one day cycle

Supplementary Table 3: Cluster map of gene signature affected by therapy and predicting response

Flt3ITD	+	-	+	+	-	+	+	+	+	+	-	-	-	-	+/TKD
Sor/Vor<br><br>Sor/Vor/Bor<br>(Coded Blue in the Row)	#7<br>VGPR	#8<br>CR	#14<br>VGPR	#9<br>NE/+PB	#11<br>PR	#5<br>CRi	#6<br>CRi	#12<br>PR	#13<br>CRi	#17<br>CRi	#4<br>NR	#6<br>NR	#15<br>NR	#4<br>NR	#7<br>NR
DAPK1	-	-		-	-	-	-	-	-	-	-	-	-	-	-
RUNX3	-						-						-	-	
CDKN2A	-			-	-	-	-		-	-	N/A	N/A		-	-
ID1				-	-			-			N/A	N/A	-	-	-
MEIS1		-	-		-	-		-			-	-		-	-
JUN			-		-			-	-	-	-		-	-	-
PIM1		-	-	-	-	-		-	-	-	N/A	N/A		-	-
PIM2		-	-	-	-	-		-		-	N/A	N/A		-	-
HOXA9			-		-			-			-	-		-	-
HOXA10		-	-		-			-		-	-	-		-	-
TET2[a]	0.65<br>M	1.0	0.35	0.6	0.75	0.39	0.65<br>M	0.3<br>M	0<br>M	0.1<br>M	N/A	1.25	N/A	1.27	3.1

a- hypomorphic Tet2 quantitative levels colored blue; M= mutant profile

Red box: gene significantly upregulated by therapy

Blue box: gene significantly downregulated by therapy

Orange box: gene upregulation with borderline significance

N/A: not available
